# Direct Observation of Enzymes Replicating DNA Using a Single-molecule DNA Stretching Assay

**DOI:** 10.3791/1689

**Published:** 2010-03-23

**Authors:** Arkadiusz W. Kulczyk, Nathan A. Tanner, Joseph J. Loparo, Charles C. Richardson, Antoine M. van Oijen

**Affiliations:** Harvard Medical School

## Abstract

We describe a method for observing real time replication of individual DNA molecules mediated by proteins of the bacteriophage replication system. Linearized λ DNA is modified to have a biotin on the end of one strand, and a digoxigenin moiety on the other end of the same strand. The biotinylated end is attached to a functionalized glass coverslip and the digoxigeninated end to a small bead. The assembly of these DNA-bead tethers on the surface of a flow cell allows a laminar flow to be applied to exert a drag force on the bead. As a result, the DNA is stretched close to and parallel to the surface of the coverslip at a force that is determined by the flow rate (Figure 1). The length of the DNA is measured by monitoring the position of the bead. Length differences between single- and double-stranded DNA are utilized to obtain real-time information on the activity of the replication proteins at the fork. Measuring the position of the bead allows precise determination of the rates and processivities of DNA unwinding and polymerization (Figure 2).

**Figure:**
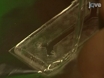


## Protocol

### 1. DNA Replication Template

DNA for the reaction is a linearized λ DNA modified by annealing of oligonucleotides to form a replication fork.  Additionally, biotin is attached to the end of one strand of the λ DNA, and a digoxigenin moiety is attached to the other end of the same strand^1^.

**Materials: ** Bacteriophage λ DNA, Oligonucleotides: biotinylated fork arm (A: 5'-biotin-AAAAAAAAAAAAAAAAGAGTACTGTACGATCTAGCATCAATCACAGGGTCAGGTTCGTTATTGTCCAACTTGCTGTCC-3'), λ-complementary fork arm (B: 5'-GGGCGGCGACCTGGACAGCAAGTTG GACAATCTCGTTCTATCACTAATTCACTAATGCAGGGAGGATTTCAGATATGGCA-3'), fork primer (C: 5'-TGCCATATCTGAAATCCTCCCTGC-3'), λ-complementary digoxigenin end (D: 5'-AGGTCG CCGCCCAAAAAAAAAAAA-digoxigenin-3'), T4 DNA Ligase, T4 polynucleotide Kinase, Heat block.

Phosphorylate the 5' ends of oligonucleotides B and D using T4 Polynucleotide Kinase.
Anneal oligonucleotides A, B, and C to the λ DNA in one step by adding a 10-fold excess of oligonucleotides A and B, and a 100-fold excess of oligonucleotide C in T4 DNA ligase buffer.
Heat the mixture to 65 °C in the heat block. Once heated, turn the heat block off and allow slow cooling to properly anneal oligonucleotides.
Add T4 DNA ligase to the mixture and incubate at room temperature for 2 hours.  T4 DNA Ligase will catalyze the correct joining of oligonucleotides A and B, and λ DNA.
Finally, anneal the oligonucleotide D to the DNA replication template by adding 100-fold excess of oligonucleotide D with respect to l DNA in T4 DNA ligase buffer.
Incubate the mixture at 45 °C for 30 min in the heat block, and cool to room temperature slowly by turning heat block off.
The final DNA replication template is now ready for use.  We store the template at a concentration of 1.4 nM at 4 °C for several weeks.

### 2. Beads

Beads are functionalized with a Fab fragment with specificity for digoxigenin. They can be then attached to the DNA replication template^2^.

**Materials: **Tosyl activated beads, α-digoxigenin Fab fragment, Buffer A: 0.1 M H3BO3 pH 9.5, Buffer B: 0.1 M PBS pH 7.4, 0.1% w/v BSA, Buffer C: 0.2 M Tris-HCl pH 8.5 (25 °C), 0.1% w/v BSA, Magnetic Separator, Rotator.

Resuspend the stock solution of beads and transfer a small aliquot into the eppendorf tube. Place the tube into a slot of the magnetic separator and incubate until solution clears, then remove supernatant by pipetting.
Add buffer A, which will activate tosyl groups for antibody coupling. Mix gently by pipetting and remove buffer with the magnetic separator.
Add Fab fragment and resuspend beads again in buffer A, mix thoroughly, and incubate 16-24 hours at 37 °C using rotator. After incubation, separate beads using the magnetic separator and remove buffer.
Wash beads by adding buffer B and incubate at 4 °C for 5 min. Remove the buffer using magnetic separator.  Repeat washing twice.
Add buffer C and incubate at 37 °C for 4 hours to block free tosyl groups. Separate beads using magnetic separator and remove buffer.
Wash twice in buffer B and aliquot for use. Beads can be stored at 4 °C for 1 year or longer without substantial loss of quality (our stock solution contains 1- 2x10^9^ beads/ml).

### 3. Functionalized Coverslips

To allow attachment of the DNA to the glass coverslip, the glass is first functionalized with an aminosilane, which is then coupled to biotinylated PEG molecules. This coating helps to reduce the nonspecific interactions of DNA and replication proteins with the surface^3,4^.

**Materials:** Glass coverslips, Staining jars, 3-aminopropyltriethoxysilane, Methoxy-PEG5k-NHS ester, Biotin-PEG5k-NHSester, Acetone, 1M KOH, Ethanol, Oven, Bath sonicator

Thoroughly clean the glass coverslips by placing them in staining jars and filling the jars with ethanol. Sonicate for 30 minutes, rinse out the jars and fill with 1 M KOH. Repeat both washes.
For the first functionalization reaction, all traces of water need to be removed. Fill the jars with acetone and sonicate for 10 min. Empty and fill again with acetone.
Prepare a 2 % v/v solution of the silane reagent in acetone. Add to the staining jars and agitate for 2 minutes. This reaction couples the alkoxy group of the aminosilane to the glass, leaving a reactive amine for the next coupling step.  Quench the reaction with a large excess of water.
Dry the coverslips by baking them at 110 °C in the oven for 30 minutes.
Prepare a 50:1 methoxy:biotin PEG mixture in 100 mM NaHCO_3_ pH 8.2. Aim for around 0.2 % w/v biotinylated PEG.
Pipette 100 μL of the PEG mixture onto a dry silanized coverslip and place another coverslip on top. Including a glass coverslip spacer will allow separation of the coverslips.
Incubate the coverslips in the PEG solution for 3 hours, then separate each pair of coverslips and wash extensively with water. Be careful to keep the coverslips functionalized side up as only once side will be coated with PEG.
Dry the coverslips with air and store under vacuum in desiccator. The surfaces remain stable for at least a month, so dozens of coverslips can be made in a batch and used as needed.

### 4. Flow Chamber Preparation

The experiment is performed using a simple flow chamber constructed with a functionalized coverslip, double-sided tape, a quartz slide and tubing. One flow chamber is prepared for each single-molecule experiment^2,4^.

**Materials:** Double-sided tape, razor blade, quartz slide with holes for tubing, quick-dry epoxy, Functionalized coverslip, streptavidin solution (25 μL of 1 mg/mL in PBS), tubing, blocking buffer (5X: 20 mM Tris pH 7.5, 2 mM EDTA, 50 mM NaCl, 0.2 mg/mL BSA, 0.005% Tween-20), working buffer (1X: 20 mM Tris-HCl pH 7.5, 2 mM EDTA, 50 mM NaCl).

Begin by mixing and placing 5 mL of blocking buffer and 20 ml of working buffer in a desiccator to remove any air bubbles for later steps.
Take a PEG-functionalized coverslip and spread 125 of PBS-diluted streptavidin solution over the surface. Leave this to incubate for 20 minutes at room temperature while preparing other parts of the chamber, allowing streptavidin to bind surface biotins.
Cut a piece of double-sided tape to match the size of the quartz slide. Mark the position of the tubing holes on the tape so that an outline of the flow chamber can be drawn on the tape.
Make a 3 mm-wide center channel and rectangles containing both holes. We use flow chambers with two inlet and two outlet Y-shaped channels.
Cut along the drawn outline, making straight, clean cuts to make sure no adhesive protrudes into the channel.
Clean the quartz thoroughly using acetone or ethanol to remove adhesive from the previous flow cell construction.
Find the best alignment of the channel outline, remove one side of the adhesive backing and carefully place the tape onto the quartz slide. Be careful to align the tape properly, as the inlet and outlet holes need to remain unblocked.
Cut lengths of tubing for the inlet and outlet of the flow cell. It helps to cut the end of the tube at about a 30° angle so that the tube will not press flat against the chamber bottom. Place the tubes on some sort of support (e.g. tube racks) to suspend them for easy attachment to the flow cell in the next steps.
Rinse the streptavidin-coated coverslip thoroughly with water and dry using compressed air. Remember that only one side is functionalized. Remove the other side of the tape backing and place the quartz slide onto the coverslip.
Lightly press on the coverslip to push out any air trapped in the adhesive. This will help avoid any air bubbles getting into the flow channel.
Seal the sides of the chamber with epoxy. Insert the cut tubing into the holes of the quartz and seal in place with epoxy. This needs to dry for a few minutes.
Once dry, begin blocking the surface by pulling some of the degassed blocking buffer through one of tubes. A 21-gauge needle fits perfectly inside the 0.76 mm tubing. Flush out a few times to remove air bubbles, and allow the chamber to incubate for at least half an hour.

### 5. Single-molecule Replication Experiment

Once the DNA template and functionalized beads have been prepared, and the flow chamber is ready, a single-molecule experiment can be performed.

**Materials:** Prepared flow chamber, Blocking buffer (5X : 20 mM Tris-HCl pH 7.5, 2 mM EDTA, 50 mM NaCl, 1 mg/mL BSA, 0.025% Tween-20), Working buffer (1X: 20 mM Tris-HCl pH 7.5, 2 mM EDTA, 50 mM NaCl), T7 replication buffers with or without 10mM MgCl_2_ (1X: 40 mM Tris-HCl pH 7.5, 50 mM K-glutamate, 2 mM EDTA, 100 μg/ml BSA, 10 mM DTT, 600 μM dNTPs), replication proteins, inverted optical microscope with 10X objective, CCD camera, permanent rare-earth magnet, syringe pump, airspring, fiber illuminator, computer with image acquisition software.

After the flow cell has been blocked, it is ready to begin the experiment.
Take the flow cell and place it on the microscope stage. Hold the chamber in place with stage clips and be sure that the flow channel is positioned in alignment with CCD camera.
Connect the flow cell outlet tubes to the airspring using a larger diameter connector tubing or a needle. The airspring is used to stabilize any flow irregularities. A 50 ml plastic tube is filled with 45ml of water. The lid of the tube is sealed with epoxy. Three holes are pierced in the lid, and three pieces of tubing are inserted into the tube. Using epoxy, the tubes are then sealed to the lid, preventing any air from entering or escaping. The airspring is connected to the syringe pump, and to the outlet tubes of the flow cell.
Place the inlet tubes of the flow cell in blocking buffer and pull back on the syringe to remove any air in the tube. A gentle flick of the outlet tube will help clear any air bubbles trapped in the flow cell.
Once all air bubbles have been removed, block one of the inlet tubes with a needle, and one of the outlet tubes by bending it by 1800 and securing the tube with adhesive tape.
Dilute the stock DNA template to 10 pM in 1 ml of degassed blocking buffer. Flow into the chamber at moderate flow rate to allow good surface coverage of DNA (0.01-0.05 ml/min for 20 minutes works well). This can be varied based on how many DNA molecules are on the surface for each batch of coverslips or how many are desired for the experiment.
Dilute the stock bead solution to 2-4x10^6^ beads/ml in 1 ml of degassed blocking buffer. Flow into the chamber at 0.01-0.05 ml/min for 15 minutes.
Once beads are added, turn the flow off and allow chamber to reach equilibrium. Then close both outlet tubes. Any changes to the tubes without the chamber closed will cause pressure fluctuations that will exert a strong force on the beads and shear the tethered DNA molecules.
Block the inlet tube that was used for loading beads with a needle, and begin washing the flow cell using the second inlet with degassed 1X solution of blocking buffer in working buffer. Wash extensively at the rate of 0.01-0.05 ml/min. Agitate manually the stage by tapping to remove any beads nonspecifically stuck to the surface (Tapping).
Once free beads are sufficiently removed, turn the flow off and allow chamber to reach equilibrium. Close the open outlet tube and switch the inlet reservoir to protein solution, slowly opening the outlet to avoid rapid pressure changes
You can check if beads are tethered to DNA by changing direction of the flow (DNA).
Now, the enzymatic reaction can be performed. For leading-strand synthesis experiments make 20nM solution of gp4 helicase and 20nM solution of gp5 polymerase (purified as a complex with its processivity factor thioredoxin) in degassed T7 replication buffer that does not contain MgCl_2_.
Flow the protein solution into the chamber at moderate flow rate to allow efficient binding to DNA.  We use 0.037 ml/min for 8 minutes, and then 0.012 ml/min for 7 minutes.
Wash the chamber with degassed T7 replication buffer without MgCl_2_ to remove free proteins. The rate of 0.037 ml/min for 3-10 minutes works fine.
After washing, flow T7 replication buffer with MgCl_2_ and begin data acquisition. Use a flow rate of 0.012 ml/min corresponding to 3 pN drag force on the DNA tethers under conditions described in this protocol.
Place a rare-earth permanent magnet above the flow cell before imaging to prevent nonspecific interactions between beads and surface.
View field with a 10X objective and CCD camera. Focus using a tethered bead.
Position a fiber illuminator at an incidence angle between 10 degrees and parallel to the microscope stage near the microscope. Dark-field illumination is used to increase the contrast in the bead imaging.
Acquire data for 20 30 min at a slow frame rate (typically 2-4 Hz).

### 6. Representative Results

In a successful experiment you should be able to observe more than 100 beads simultaneously (Leading strand synthesis). The experiment should yield numerous traces displaying leading strand DNA synthesis.

#### Data Analysis:

In order to measure rates and processivities of DNA unwinding and polymerization, the precise positions of beads must be determined. You can achieve it by fitting these positions with 2-dimensional Gaussian functions. Trajectories can then be extracted by tracking bead position at each frame using tracking software (e.g. DiaTrack). Now you are ready to visualize trajectories by plotting bead position as a function of time using any graphic software (e.g. Origin). To obtain rate data, fit the plots with a linear regression and calculate the slope. For processivity, determine the total length of the DNA from start to end of a shortening event (Figure 3). Both of these numbers will need to be converted to basepairs. To convert bead movement to basepairs, first you need to measure the length of a stretched λ DNA molecule at the reaction flow rate. Since the length of the λ DNA is known (48,502 bp) you can calibrate the number of base pairs/pixel at experimental force. For increased accuracy, subtract from the traces of interest a baseline trace of a bead tether that is not enzymatically altered. Combine all single measurements and plot rate and processivity distributions. Fit the data using Gaussian and single-exponential functions, respectively (Figure 3).


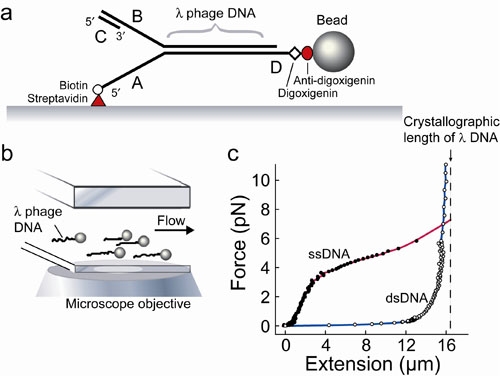
 **Figure 1. **Single-molecule experimental setup. (a) Duplex λ DNA (48.5 kb) is attached to the surface of the flow cell via the 5' end of one strand using a biotin-streptavidin interaction, and the 3' end of the same strand is attached to a bead using a digoxigenin anti-digoxigenin interaction. A primed replication fork is formed at the end opposite the bead to allow loading of the polymerase. (b) Bead-DNA assemblies are stretched using laminar flow of buffer and imaged using wide-field optical microscopy. By tracking the positions of the beads over time, while maintaining a constant stretching force, the lengths of the DNA constructs can be monitored. (c) Extension profile of ssDNA (filled circle) and dsDNA (open circle) under low forces. Dashed line shows crystallographic length of fully ds-λ DNA, 16.3 μm. The large difference in length between ssDNA and dsDNA at forces around 3 pN allows a direct observation of conversions between ss- and dsDNA by monitoring changes in the DNA length. The simultaneous visualization of large numbers of DNA-coupled beads allows for the study of many individual replisomes in one experiment.


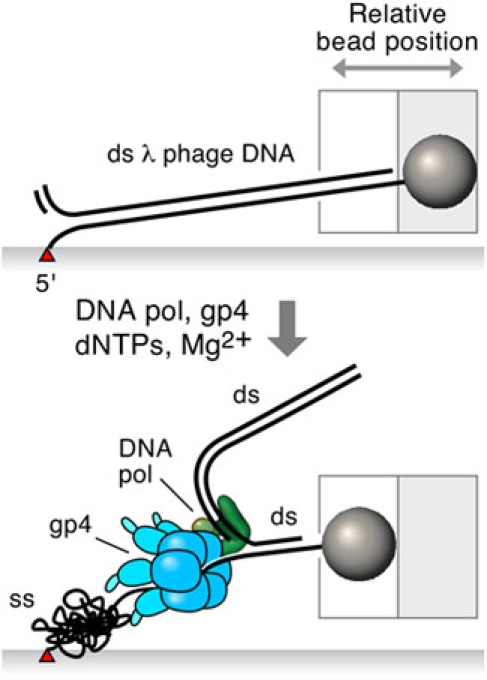
 **Figure 2. **Bacteriophage T7 replication proteins: DNA polymerase (complex of gp5 and thioredoxin) and DNA helicase (gp4) synthesize DNA leading strand in the presence of deoxyribonucleotides and magnesium. This process is accompanied by shortening of DNA lagging strand due to coiling. Conversion of dsDNA into ssDNA changes position of the bead. Measuring position of the bead allows precise determination of the rate and processivity of DNA replication.


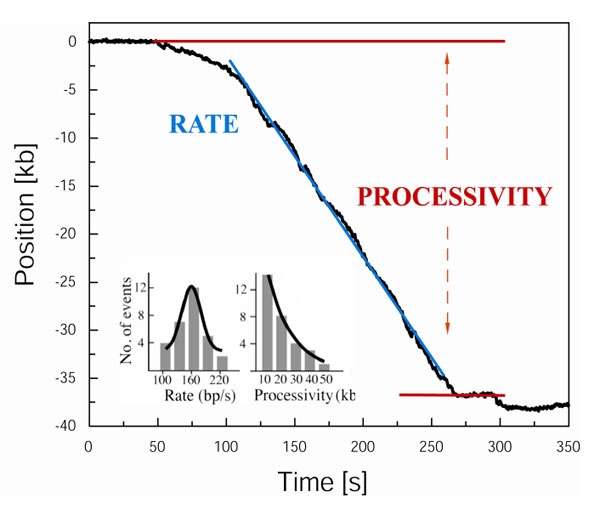
 **Figure 3. **Example of typical data from a single-molecule leading-strand synthesis experiment. Processivity of DNA replication is equal to a total length of the DNA from start to end of a shortening event. The rate of DNA replication is obtained by fitting the plot with a linear regression and calculating the slope. The inset shows rate and processivity distributions that were obtained by combining data from a number of measurements. Data were fitted using Gaussian and single-exponential functions, respectively.

## Discussion

It is important to ensure that the drag force exerted on beads by laminar flow doesn't influence enzymatic activities of the replication proteins. For instance, a 3 pN force that corresponds to a flow rate of 0.012 ml/min does not affect replication of the DNA leading strand. It does however affect enzymatic activities that take place during coordinated DNA synthesis, and it therefore has to be reduced to 1.5 pN. The drag force can be easily controlled by changing the flow rate or a width of the flow channel^5^.

The DNA stretching assay employs length differences between double- and single-stranded DNA. In the experiment described here the conversion of dsDNA to ssDNA occurs as a result of the leading strand synthesis (Figure 2). You should remember that the ssDNA is shorter than dsDNA only at low stretching forces below 6 pN (Figure 1).

A possible improvement of this method is to modify the lagging strand of the replication template by attaching a second bead to its 3'-end. Such alternation would allow direct observation of enzymatic activities that take place at both strands of DNA.

Apart from studies of the leading strand synthesis and coordinated replication, the DNA stretching assay has been successfully employed to examine the single-molecule kinetics of λ exonuclease, and dynamics of polymerase exchange during replication^5,6^. In principle, this method can be used to study any DNA processing enzyme.
